# Supporting desistance from crime and recovery from drug use with a patient-facing bidirectional eHealth tool: a case report

**DOI:** 10.1186/s13256-026-06152-2

**Published:** 2026-05-28

**Authors:** Shoresh Palanijafi, Johan Månflod, Karl Andersson

**Affiliations:** 1 Min Framtid AB, Karlstad, Sweden; 2 Aros Forskning & Friskvård AB, Uppsala, Sweden; 3 Skillsta Teknik Design Och Kvalitet AB, Vänge, Sweden; 4 IGP, Uppsala University, Uppsala, Sweden; 5 Present Address: Gottsunda vårdcentral, Region Uppsala, Uppsala, Sweden

**Keywords:** Case report, Substance use disorder, eHealth, Drug testing, Gang disengagement

## Abstract

**Background:**

Simultaneous exit from both drug use and a criminal organization is a difficult and potentially dangerous process for the individual.

**Case presentation:**

This case report describes the use of the eHealth tool Previct Drugs to support a 27-year-old Caucasian male with substance use disorder in exiting an organized crime gang and achieving both sobriety and a law-abiding lifestyle. The eHealth tool is a smartphone application that provides eye-scanning-based drug screening to promote sobriety, as well as cognitive behavioral therapy and motivational interviewing. A strong therapeutic alliance was rapidly established, supported by continuous insight into the individual’s sobriety and mood through the eHealth tool. The individual used the system for 7 months.

**Conclusion:**

Care could be delivered with precise timing when the individual signaled a need for support, preventing at least one relapse. According to caregiver estimates, use of the eHealth tool accelerated the process of achieving sobriety and exiting the criminal lifestyle by several months.

## Background

Members of criminal organizations and street gangs are associated with armed violence [1], illicit drug use [2], and other traumatic events in addition to the criminal activities targeting those outside the organized crime gang. The process of exiting criminal organizations or street gang is a difficult and dangerous procedure for the individual, in particular if the individual has a concurrent use of illicit drugs. Furthermore, in contrast to the recruitment process, few have explored disengagement from a research perspective [3].

In their review of disengagement of street gangs, Tonks and Stephenson [3] describe the process of exiting an organized crime gang as complex and multifaceted. The role of significant others is emphasized. Whereas the term “significant others” often is understood as a family member, the concept extends to also include care staff who form a bond with the person about to exit gang life, similar to but stronger than a therapeutic alliance [4]. Other contributing personal factors for initiating an exit from an organized crime gang include parenthood, self-reflection, and maturation. Perception aspects such as feelings of victimization and disillusionment may also be part of an exit decision, as may the physical removal of oneself from the gang environment, through criminal sanction for example [3].

Among the plethora of medical grade health apps, few or even none address the issue of substance use disorder. Research on eHealth for substance use disorder management is, however, vibrant and active [5]. This case report describes the acceleration of creating a strong therapeutic alliance with an individual in the exit from both organized criminality and drug use through use of an eHealth tool, Previct Drugs. This tool is an app capable of providing eye-scanner-based drug screening to promote sobriety, as well as cognitive behavioral therapy (CBT), and motivational interviewing (MI). While the client utilizes the Previct Drugs app on their smartphone to perform tests and log daily activities, healthcare providers monitor this data through the Previct Care Portal. The 24/7 support provided to the individual through this digital tool, literally available in his pocket, is described and discussed.

## Case presentation

This case report is authored in compliance with CARE Case Report Guidelines [6]. The case report relates to a Caucasian male, 27 years old, who was in the process of exiting a criminal organization. The individual had an illicit substance use pattern of primarily amphetamine, but also heroin and cannabis. He had been in and out of drug rehabilitation many times in recent years. Like many other members of criminal organizations [7], the individual is diagnosed with post-traumatic stress disorder (PTSD).

The individual was provided with the smartphone-based eHealth tool Previct Drugs (Kontigo Care AB). This report was authored 7 months after initiation, in conjunction with terminating the use of Previct Drugs. Previct Drugs is both an AI-based eye-scanning-based drug testing system [8] [9] and a support platform where individualized treatment programs, CBT, MI, and a help-button, which signals an emergency and need for help, can be combined for assisting substance use disorder patients back to sobriety. The platform requires a mid-range smartphone or better to function.

Alongside access to the eHealth tool, care as usual was provided through caregiver–patient consultations three to four times per week. The weekly urine-based drug testing (LITA Multi-15, Prodia, Vellinge, Sweden) conducted before initiation of the eHealth tool was continued when the eye-scanning-based drug indication was in place.

## Results

From the perspective of the individual, use of the eHealth tool brought many advantages. The drug testing frequency increased from twice per week (urine-based) to twice per day (eye-scanning based). The frequent and location-independent drug testing brought many advantages. First, each conducted test that reported a result “sober” became an encouragement to the individual, a confirmation that he could stay sober and a reason for declining drug use. Second, the inconveniences of urine-based testing, both with the transportation of oneself to the clinic and with urination under observation of clinical staff, vanished entirely. Using the words of the individual:

*You see yourself on your mobile phone and a voice tells you what to do to scan your eyes. I had to do the test twice a day. You got into it pretty quickly. It’s convenient and much better than going in to do urine tests twice a week, which is very stressful. It is not always possible to pee on demand.*

Third, the eHealth tool contains several short mood- and motivation-related questionnaires to track risk and well-being factors, identifying early signs of relapse. The eHealth tool also includes an individualized care program, offering support for new habits and CBT exercises. Assignments were used to enhance the face-to-face treatment by providing new insights to address in caregiver–patient consultations while in parallel supporting new positive habits and traits on a daily basis. These enable communication of feelings and conditions also for those who have difficulty expressing feelings or difficulty writing. In the case of the individual, the questionnaires helped to convey how he was feeling to the care provider. One questionnaire related to honesty was particularly important to the individual. Using the words of the individual:

*I have a hard time talking about how I feel, so the app really helps.*

*It has helped me reflect on honesty and reminds me to be so when I go to meetings and so on, that I dare to talk more.*

From a caregiver perspective, use of the eHealth tool also brought many advantages in this case. The eHealth tool does not replace the caregiver–patient consultations, but it makes each consultation more efficient. Since the caregiver has immediate information about what has happened between consultations, the discussion can instead focus on the individual in the here and now. As an example, the eHealth tool aids the individual in expressing and understanding feelings in advance of the consultation, so if it has been a week of bad sleep, it is known upfront and allows ample time to discuss remedies during the consultation. This was valuable support, accelerated the formation of a strong therapeutic alliance, and shortened the care process.

Withdrawal of amphetamine generally introduces risk for fatigue, depression, anxiety irritability, mood swings and strong craving for drugs, these symptoms in addition to the complicated social pressure were associated with organized crime gang disengagement. The use of cannabis and heroin were, for this individual, a lesser problem compared to other complications. The symptoms of withdrawal from amphetamine observed in this particular case were dominated by anxiety and craving, and sometimes required assistance at short notice. The individual pressed the help-button four times when in alarming need for support, which immediately alerted the caregiver to contact the individual by phone. Through a 20–30 min phone call with the individual, the caregiver could identify the problem and help the individual reach a point where the craving or anxiety was manageable. At least one very likely drug relapse was prevented through the just-in-time adaptive intervention guided by help-button use.

After 3 months, the individual reached a level that normally requires 9 months of work. The individual was confirmed drug-free during the entire period and discontinued drug screening after 7 months but continues with access to CBT, MI, and the help button in the eHealth tool. In conjunction with the discontinuation of drug screening, the individual was admitted to an outpatient psychiatry ward, where at least 3 months confirmed drug sobriety is required.

Findings from urine-based testing matched findings from eye-scanning tests. No adverse events or unanticipated events related to the eHealth tool were reported.

## Discussion and conclusion

Organized crime is a significant problem in society. Both preventing recruitment and supporting those exiting such an organization should be prioritized. This report focuses on the use of a new eHealth tool for supporting one individual in the process of exiting an organized crime gang, in terms of both drug sobriety and general care efficiency.

There are a number of health risks related to substance use disorders, especially when injected. The need for tools to combat these disorders can never be emphasized enough. Drug use presents many health hazards and in some cases it is a matter of life or death. Drug dependence often leads to social problems and criminality, thus affecting family, friends, and the whole society. Exiting a criminal organization while at the same time withdrawing from substance use disorder is, therefore, a true challenge where support from society is critical for success while also being economically sound: Every dollar invested in substance abuse treatment programs is estimated to provide 4–7 dollars in return only in reduced crime, and at least 12 dollars if healthcare savings are added [8].

Support systems that are available on smartphones have an unprecedented reach. Available literally in the user’s hand, 24/7, such systems may completely alter the view of support and care of certain groups, such as fragile individuals with mental health problems or in this case someone leaving an organized crime gang. The clinical experiences shared in this case report related to exiting substance use disorder agree with discussions in Marsch [5] and Perski et al. [10]. The awareness that digital support is immediately available and the knowledge that drugs are tested for frequently serve as a reason to resist temptation, and should the temptation be unmanageable, just-in-time adaptive interventions can be provided. The individual described in this report responded well to the digitally connected, immediately reactive care.

From a general standpoint, the ability to provide just-in-time adaptive interventions makes it possible to avoid relapses that could keep the patient in continued drug dependence and with that a need for the connection with the criminal environment. There is also a major risk of a lethal overdose after detoxification, when the drug sensibility is back to normal after the increased tolerance that comes with chronic drug use. The most dangerous drugs are the opioids, where a heroin or fentanyl overdose can result in breathing depression, respiratory acidosis, and circulatory failure. Smartphones present an ideal platform for just-in-time adaptive interventions, and the impact could be massive.

The organized and frequent collection of data is made possible by eHealth support systems, which in turn is the foundation for both efficiency in consultation and for enabling modern data analytical tools [5]. In the short term, as seen in the case described in this report, immediate access to self-reported mood and honesty accelerated and empowered the caregiver–individual relationship to form a strong therapeutic alliance in a short time. In this case, several months of counseling were saved. In the long term, collected data may result in predictive tools, such as the relapse predictor in Previct Alcohol [11].

The implementation of eHealth for supporting vulnerable patients has been discussed in the research community [5] [10] but reports from clinical implementation are lacking. This report is limited to observations related to a single case using one type of eHealth tool. Our findings related to the simultaneous exit process from organized crime gangs and recovery from substance use disorder are, however, such that a more detailed and organized clinical evaluation of eHealth tools in this context is warranted.

## Data Availability

All data generated or analyzed during this study are included in this published article.
